# When it affects me: the role of perceived media influence on self and others in supporting regulation of health misinformation

**DOI:** 10.3389/fpsyg.2026.1695690

**Published:** 2026-02-20

**Authors:** Mihee Kim

**Affiliations:** Department of Media and Communication, Sejong University, Seoul, Republic of Korea

**Keywords:** COVID-19, health communication, health misinformation, health policy, information regulation, presumed media influence

## Abstract

Guided by the Influence of Presumed Media Influence model, this study investigated how exposure to health misinformation shapes public support for regulating such misinformation on social media in the context of COVID-19. An online survey was conducted in South Korea (*N* = 400), and the data were analyzed using a moderated mediation model with the SPSS PROCESS macro. Results revealed that exposure to COVID-19 misinformation significantly increased perceptions of its influence on others. However, perceived influence on others alone did not predict support for regulation. Instead, the indirect effect of exposure on regulatory support—via presumed influence on others—emerged only when individuals also perceived misinformation as personally affecting themselves. These findings underscore that public support for regulating health misinformation is strongest when misinformation is viewed as a shared health threat, endangering both individuals and the broader community. This suggests that in the context of public health crises, regulatory attitudes are driven not solely by concern for others but by the combined recognition of personal and collective risks. The study offers theoretical insights and practical implications for policymakers, health communicators, and social media platforms seeking to design policies or interventions that protect public health by countering misinformation.

## 1 Introduction

During public health crises, social media platforms function as major channels for rapid information sharing but also accelerate the spread of misinformation, as documented during the Zika, Ebola, and COVID-19 outbreaks (Gui et al., 2017; Li and Shin, 2023; Shin and Kee, 2023; Shin et al., 2023; Ghenai and Mejova, 2017; Oyeyemi et al., 2014; Al-Zaman, 2022; Apuke and Omar, 2021; Cheng and Luo, 2021; Leskin, 2020; Liu and Huang, 2020; Sun et al., 2021). Such misinformation distorts facts, provokes anxiety, and undermines effective health responses (Pew Research Center, 2016; The Independent, 2020; Mian and Khan, 2020). Although governments and health agencies have introduced measures to limit its circulation (World Health Organization, 2020a), public support for regulatory policies is often divided, particularly due to concerns about freedom of expression (Jang and Kim, 2018; Yang and Horning, 2020; Cheng et al., 2021).

To explain public attitudes toward such regulation, this study applies the Influence of Presumed Media Influence (IPMI) model (Gunther and Storey, 2003), which posits that individuals’ perceptions of media effects on others can motivate attitudinal and behavioral responses. While prior research has primarily emphasized presumed influence on others as a key mechanism driving support for regulation (Cheng and Chen, 2020), emerging work suggests that perceived influence on oneself may play a crucial role when media issues are highly personally relevant, such as in public health crises (Chung and Moon, 2016; Baek et al., 2019; World Health Organization, 2020b). However, the theoretical conditions under which perceptions of others’ susceptibility give rise to support for regulatory intervention remain insufficiently specified.

Addressing this gap, the present study proposes and tests a moderated mediation model in which the indirect effect of exposure to COVID-19 misinformation on support for regulation—via presumed influence on others—is conditioned by perceived influence on self. Rather than treating perceived self-influence as a parallel predictor, this study conceptualizes it as a critical boundary condition that determines whether presumed harm to others leads to policy support. Conceptually, the primary contribution of this study is to demonstrate that perceived influence on oneself functions as a necessary condition under which presumed influence on others translates into support for regulation in high-risk health misinformation contexts. In doing so, the study advances the IPMI framework by specifying its boundary conditions in the context of infectious disease misinformation.

By empirically examining this conditional process in the context of the COVID-19 pandemic, this study contributes to a more refined understanding of how personal risk perceptions interact with social-level judgments in shaping public support for the regulation of health misinformation. In doing so, it helps to clarify theoretical mechanisms within media-effects research and offers cautious yet relevant implications for health communicators and policymakers concerned with public trust and communication strategies during public health emergencies.

### 1.1 The presumed influence of COVID-19 misinformation

The Influence of Presumed Media Influence (IPMI) model builds on the Third-Person Perception (TPP), which holds that individuals believe persuasive media messages affect others more than themselves (Davison, 1983). This self–other asymmetry is often explained by optimistic bias, as people view themselves as less vulnerable and more discerning than the “general public” (Perloff, 1993; Gunther and Mundy, 1993; Perloff, 1996; Perloff, 2002). Unlike TPP, however, the IPMI model does not rely on such self–other comparisons. Instead, it is grounded in the Persuasive Press Inference (PPI) framework, which suggests that individuals infer public opinion by assuming that others are exposed to the same content they consume (Xu and Gonzenbach, 2008; Gunther, 1998; Cohen and Tsfati, 2009; Tal-Or et al., 2010; Huh and Langteau, 2007).

This process reflects individuals’ tendency to act as “naïve social scientists,” using limited personal experience to make broader social judgments (Eveland et al., 1999; Heider, 1958). When media content appears pervasive, people are more likely to assume it is widely disseminated and influential—a phenomenon known as the extrapolation effect (Gunther and Storey, 2003; Gunther, 1998). Following this logic, the IPMI model posits that exposure leads people to presume others are also exposed, thereby reinforcing perceptions of media influence on others (Gunther et al., 2006; Shen and Huggins, 2013; Cho et al., 2021).

A key factor in this process is the perceived social desirability of content. Research shows that socially undesirable messages (e.g., media violence) are presumed to exert stronger influence on others than desirable ones (Gunther and Mundy, 1993; Duck and Mullin, 1995). Given that health related misinformation is widely regarded as socially undesirable, individuals are likely to assume it has harmful effects on others (Cheng and Chen, 2020). Specifically, supporting evidence indicates that attention to COVID-19 misinformation predicts perceptions of others’ attention, which in turn drives presumed influence on others (Heider, 1958). Accordingly, this study predicts:

*H1*: Exposure to COVID-19 misinformation on social media will increase individuals’ presumed influence of such misinformation on others.

### 1.2 Behavioral outcomes of the presumed influence of COVID-19 misinformation

The IPMI model not only addresses perceptions of media influence but also incorporates behavioral responses as a central component (Gunther and Storey, 2003). Perceptions of media influence on others have been shown to motivate individuals to support actions aimed at mitigating the presumed negative effects of media (Sun et al., 2008). Whereas TPP research focuses on comparative judgments between the self and others regarding behavioral outcomes of media effects, proponents of the IPMI model argue that individuals’ attitudes and behaviors can be shaped solely by their perceptions of media influence on others—regardless of any self–other differential (Tal-Or and Tsfati, 2020). In other words, merely recognizing the media’s influence on others can be sufficient to elicit behavioral responses (Gunther and Storey, 2003; Tal-Or and Tsfati, 2020).

Empirical support for this model comes from Gunther and Storey’s (2003) study in Nepal, which examined the effects of a serial radio drama promoting maternal health. Although the drama was primarily intended for clinic health workers, many Nepali women were also exposed to the program. The study found that women’s attitudes toward health workers and their health-related behaviors were indirectly influenced—not through direct exposure effects, but through their perceptions of how the program influenced health workers. This finding underscores the central tenet of the IPMI model: media can exert significant indirect effects on individuals by shaping their beliefs about how others are influenced.

Since Gunther and Storey’s (2003) seminal study, a growing body of literature has validated the IPMI model in explaining its attitudinal or behavioral outcome across diverse media contexts (Cohen and Tsfati, 2009; Tal-Or et al., 2010; Huh and Langteau, 2007; Gunther et al., 2006; Hong and Kim, 2020; Ho et al., 2020a). With increasing concerns about the negative effects of misinformation circulating on social media (Yang and Horning, 2020; Yang and Tian, 2021; Ho et al., 2020b; Riedl et al., 2022; Hong, 2020), numerous studies have adopted the IPMI framework to investigate public attitudes toward regulating misinformation or fake news.

In particular, the IPMI model has been widely applied to the context of COVID-19 misinformation. For instance, Sun et al. (2021) demonstrated that perceived influence of misinformation on others elicited anticipated guilt, which, in turn, increased individuals’ willingness to correct COVID-19-related misinformation. In the same context, Shi et al. (2022) reported that individuals’ perceptions of the harmful impact of COVID-19 misinformation on others significantly predicted their intention to correct such misinformation on social media. Sun et al. (2022) also found that pro-vaccination individuals who believed others were susceptible to anti-vaccination misinformation were more likely to support regulations targeting such misinformation. Drawing upon this body of literature, the present study hypothesizes that individuals’ perceptions of the influence of COVID-19 misinformation on others will be positively associated with their support for efforts to regulate such misinformation on social media:

*H2*: Individuals’ presumed influence of COVID-19 misinformation on others will increase their support for regulations against such misinformation on social media.

### 1.3 The presumed influence of COVID-19 misinformation on self

The presumed media effect on self was not considered an essential part of this model (Gunther and Storey, 2003). Nevertheless, some studies have examined how people’s presumed media influence on themselves and others can concurrently predict their attitudes and behaviors. For example, research on blog posts after the Fukushima nuclear disaster found that perceived effects on the self significantly predicted behavior, in some cases more strongly than effects on others (Jin et al., 2018). Similarly, a meta-analysis of censorship studies showed that while presumed influence on others was the most consistent predictor, self-perceptions became more influential when issues were highly personally relevant (Schmierbach et al., 2011).

Perceptions of how misinformation influences both oneself and others can strengthen a shared sense of vulnerability and collective responsibility (Neuwirth and Frederick, 2002). From a public health perspective, this process is critical: misinformation about infectious diseases such as COVID-19 has been shown to undermine compliance with preventive measures, increase vaccine hesitancy, and erode trust in health authorities. When individuals recognize that misinformation threatens not only their own health decisions but also the safety of their community, they are more likely to support interventions aimed at mitigating its spread. In this way, perceived influence on both self and others fosters a collective orientation toward protecting public health.

Applying this reasoning, individuals who acknowledge that COVID-19 misinformation has harmful effects on both themselves and society are expected to show stronger support for regulatory actions. Such regulatory endorsement reflects not only individual concern but also a recognition of the societal need to safeguard health communication environments against misinformation.

Therefore, the following hypothesis is posited:

*H3*: The impact of individuals’ perceived influence of COVID-19 misinformation on others on their endorsement of regulations against such misinformation will be more pronounced in individuals who have a greater belief in the impact of such misinformation on themselves.

As previously mentioned, the IPMI model has primarily concentrated on the mediating role of presumed media influence on others in the connection between media exposure and people’s attitudes or behaviors (Gunther and Storey, 2003). As suggested by prior research (Baek et al., 2019), individuals are more likely to endorse regulations against COVID-19 misinformation if they believe that such misinformation affects both themselves and others (H3). This suggests that the mediating impact of the presumed influence of COVID-19 misinformation on others, as outlined by the IPMI model, might be more pronounced among those who strongly believe in the influence of such misinformation on themselves. Yet, no research has examined whether the perceived influence of COVID-19 misinformation on oneself moderates the indirect relationship between individuals’ exposure to such misinformation and their support for regulations via its presumed impact on others. Accordingly, rather than proposing a hypothesis, this study poses the following research question:

*RQ1*: Does the indirect effect of individuals’ exposure to COVID-19 misinformation on their support for regulations—through the presumed influence of the misinformation on others—vary depending on the presumed influence of the misinformation on themselves?

## 2 Methods

### 2.1 Participants and study context

This study employed an online cross-sectional quantitative approach to examine the proposed hypotheses and research question, focusing on the issue of COVID-19 misinformation circulating across various social media platforms. Given the possible exposure of social media users to such misinformation, an online survey offers a convenient means of reaching social media users. The online survey was conducted in South Korea from August 24 to August 27, 2020, during the COVID-19 pandemic. Public concern about the outbreak of COVID-19 rapidly increased in South Korea due to the packed demonstrations held in downtown Seoul on August 15, 2020, the Liberation Day (Yonhap News, 2020).

Participants were recruited from a panel of a research company in South Korea. A total of 2,538 individuals were selected from the panel through quota sampling by age, gender, and region. An invitation email was sent, and 485 participants completed the survey. The response rate was 18.74%. For recruiting actual social media users, respondents who indicated not to use social media including YouTube, Facebook, Twitter, or Kakao-Talk^1^ in the last week were excluded. Those who did not finish the survey were also excluded. No post-stratification weighting was applied, as the study prioritized theoretical relationships over population estimates. This process resulted in a sample of 400, which, according to results of a G*Power analysis, is an effective sample size for subsequent statistical analyses (Faul et al., 2009). Among a total of 400 participants, 50% were female (*n* = 200) and 50% were male (*n* = 200). The average age of the respondents was 39.7 years (*SD* = 12.02). Most participants had BA degrees (*N* = 274; 69%). Their average monthly household income was between US$3,600 and 4,500.

### 2.2 Ethical statements

The study was conducted in accordance with the Declaration of Helsinki, and approved by the Institutional Review Board of Sejong University, Seoul, South Korea (SJU-HR-E-2020-18, 20 August, 2020). Informed consent was waived by the Institutional Review Board of Sejong University, Seoul, South Korea (SJU-HR-E-2020-18, 20 August, 2020). Because the study posed minimal risk to participants and the public, did not involve the collection of personally identifiable information, and did not target vulnerable populations, the requirement for informed consent was waived.

### 2.3 Measurements

#### 2.3.1 Exposure to COVID-19 misinformation on social media

Consistent with previous studies (Park, 2005), a single item measured participants’ exposure to COVID-19 misinformation on social media: “How often have you seen COVID-19 misinformation on social media. Misinformation here refers to social media content that are intentionally false and could mislead readers.” Answers were assessed on a 7-point scale from 1 (*never*) to 7 (*very often*) (*M* = 4.02, *SD* = 1.56).

#### 2.3.2 Presumed influence of COVID-19 misinformation on others and self

Presumed influence of COVID-19 misinformation on others and self were measured with statements adapted from the prior research (Baek et al., 2019; Park, 2005). Participants were directed to indicate their agreement on a 5-point scale from 1 (*strongly disagree*) to 5 (*strongly agree*) with the following three statements: “COVID-19 misinformation on social media attracts others’ (my) attention,” “COVID-19 misinformation on social media is persuasive to others (me),” “COVID-19 misinformation on social media influences others (me).” Responses showed a high reliability and were averaged to produce an index of presumed influence of COVID-19 misinformation (for others, Cronbach’s alpha = 0.76, *M* = 3.48, *SD* = 0.72; for self, Cronbach’s alpha = 0.94, *M* = 2.31, *SD* = 1.04).

#### 2.3.3 Regulations against misinformation on social media

Based on Jang and Kim (2018), participants’ support for regulations against COVID-19 misinformation on social media was measured using the following statements: “COVID-19 misinformation on social media should be banned,” “I support legislation to prohibit COVID-19 misinformation on social media,” “COVID-19 misinformation on social media should be regulated by social media platforms such as Facebook,” “COVID-19 misinformation on social media should be regulated by the government.” The items were assessed with a 5-point scale from 1 (*strongly disagree*) to 5 (*strongly agree*). Responses achieved a high reliability. Thus, an index of regulations against misinformation on social media was calculated by averaging the responses (Cronbach’s alpha = 0.91, *M* = 4.21, *SD* = 0.89).

#### 2.3.4 Analysis

**Figure 1:**
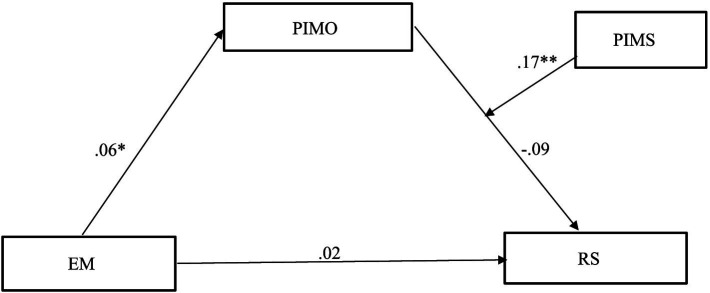
The moderated mediation effect of exposure to COVID-19 misinformation on support for regulations against such misinformation on social media. EM, Exposure to Misinformation; PIMO, Presumed Influence of Misinformation on Others; PIMS, Presumed Influence of Misinformation on Self; RS, Regulation Support. Unstandardized regression coefficients. *p < 0.05, **p < 0.001.

All analyses were conducted using the SPSS PROCESS macro (Hayes, 2013; Hayes, 2015; Preacher and Hayes, 2008). PROCESS Model 14 was conducted to test a moderated mediation model (see Figure 1). The analyses computed 95% bias-corrected confidence intervals (CIs) based on 5,000 bootstrapping samples (Hayes, 2013). The exposure to COVID-19 misinformation on social media was included as an independent variable, while the endorsement of regulations targeting COVID-19 misinformation on social media was set as a dependent variable. Additionally, the assumed impact of COVID-19 misinformation on others served as a mediating variable. The perceived influence of COVID-19 misinformation on self was introduced as a moderating variable. Demographic factors including age, gender, education, and monthly household income were entered as control variables in the analysis. The correlation of control variables with key variables is presented in Table 1.

**Table 1:** Correlation matrix of variables (*N* = 400).

Variables	1	2	3	4	5	6	7	8
1. Age	1				–			
2. Gender	−0.04	1						
3. Education	−0.04	−0.09	1					
4. Income	0.09	0.02	0.23**	1				
5. EM	−0.03	−0.10*	−0.004	0.03	1			
6. PIMO	−0.18**	0.07	0.05	−0.05	0.12*	1		
7. PIMS	−0.12*	0.08	0.04	−0.03	0.27**	0.28**	1	
8. RS	0.05	0.02	−0.11*	−0.01	0.003	0.16**	−0.07	1

EM, Exposure to Misinformation; PIMO, Presumed Influence of Misinformation on Others; PIMS, Presumed Influence of Misinformation on Self; RS, Regulation Support. * < 0.05; ** < 0.01.

## 3 Results

H1 expected that the exposure to COVID-19 misinformation would increase the perceived effects of such misinformation on others. As shown in Table 2, the results show that the more people were exposed to COVID-19 misinformation on social media, the more they believed the misinformation influenced others (*b* = 0.06, *SE* = 0.02, *t* = 2.53, *p* < 0.05). Therefore, H1 was supported.

**Table 2:** Regression results from the moderated mediation model (PROCESS model 14) for predicting the presumed influence of COVID-19 misinformation on others and support for regulations against such misinformation on social media.

Variables	PIMO	RS
	*b* (*SE*)	*b* (*SE*)
Control variables		
Age	−0.01 (0.003)***	0.01 (0.004)
Gender	0.14 (0.08)	0.03 (0.09)
Education	0.05 (0.04)	−0.13 (0.05)**
Income	−0.02 (0.02)	0.0001 (0.02)
Conceptual variables		
EM	0.06 (0.02)*	0.02 (0.03)
PIMO		−0.09 (0.11)
PIMS		−0.78 (0.05)***
PIMO × PIMS		0.17 (0.05)***
Constant	3.78 (0.25)	4.92 (0.51)
*R* ^2^	0.06	0.08
*N*	400	400

b, unstandardized regression coefficients; SE, standard error; EM, Exposure to Misinformation; PIMO, Presumed Influence of Misinformation on Others; PIMS, Presumed Influence of Misinformation on Self; RS, Regulation Support. Gender: 0 = male, 1 = female. *p < 0.05, **p < 0.01, ***p < 0.001.

H2 predicted that the presumed influence of COVID-19 misinformation on others would increase public support for regulations against such misinformation on social media. The effect of the presumed influence of COVID-19 misinformation on others on support for regulations against such misinformation was not significant (*b* = −0.09, *SE* = 0.11, *t* = −0.86, *p =* 0.*39*). Thus, H2 was disconfirmed.

H3 expected that the impact of the presumed influence of COVID-19 misinformation on others on support for regulations against such misinformation on social media would be stronger among those with higher levels of the presumed effect of such misinformation on self. The results revealed a significant interaction effect between the presumed influence of COVID-19 misinformation on others and self (*b* = 0.17, *SE* = 0.05, *t* = 3.38, *p* < 0.001). The positive relationship of the presumed influence of COVID-19 misinformation on others with public support for regulations against such misinformation on social media was significant for those who scored 1 *SD* above the mean of the presumed effect of COVID-19 misinformation on self (*b* = 0.48, *SE =* 0.10, *p* < 0.001, 95% CI = [0.289, 0.677]) and those at the mean (*b* = 0.30, *SE =* 0.06*, p <* 0.001, 95% CI = [0.181, 0.419]). This effect was not significant for those who scored 1 *SD* below the mean (*b* = 0.11, *SE =* 0.07, *p >* 0.05, 95% CI = [−0.016, 0.240]). Therefore, H3 was confirmed.

RQ1 examined whether and how the indirect impact of individuals’ exposure to COVID-19 misinformation on their support for regulations against such misinformation via the presumed effect of the misinformation on others would vary by their presumed influences of the misinformation on themselves. A moderated mediation index developed by Hayes (2015) (index = 0.01, Boot *SE* = 0.01, 95% CI [0.001, 0.025]) indicated that the indirect relationship between exposure to COVID-19 misinformation and public support for regulations against such misinformation on social media varied depending on the perceived effect of COVID-19 misinformation on self. Specifically, for those who scored 1 *SD* above the mean of the presumed effect of COVID-19 misinformation on self (*b* = 0.03, *SE =* 0.02, 95% CI = [0.002, 0.066]) and those at the mean (*b* = 0.02, *SE =* 0.10, 95% CI = [0.001, 0.041]), a positive indirect relationship between exposure to COVID-19 misinformation and support for regulations against such misinformation on social media was significant. However, the indirect relationship was not significant among those who scored 1 *SD* below the mean (*b* = 0.01, *SE =* 0.01, 95% CI = [−0.001, 0.021]). A significant direct effect of people’s exposure to COVID-19 misinformation on their support for regulations against such misinformation was not found (*b* = 0.02, *SE* = 0.03, *t* = 0.76, *p =* 0.45).

## 4 Discussion

In line with the IPMI model, the present study found that participants’ exposure to COVID-19 misinformation on social media increased their perceptions of its influence on others. However, in contrast to the model’s original premise, the perceived influence of COVID-19 misinformation on others did not independently predict support for regulations against it on social media. Instead, this relationship emerged only when individuals also believed that misinformation could personally affect themselves.

These findings offer both theoretical and practical implications. First of all, the non-significant effect of presumed influence on others on regulatory support suggests a context-dependent refinement of the IPMI framework. Attribution theory (Ross, 1977) suggests that responses to social problems can depend on how responsibility for harm is assigned. When the spread of misinformation is attributed to systemic failures such as platform algorithms, individuals are more likely to view institutional regulation as an appropriate corrective response.

In the context of health misinformation, however, individuals may attribute others’ vulnerability to dispositional factors, such as limited health literacy or poor individual decision-making. Under this internal attribution, the effect of misinformation on others can be construed as an individual-level failure rather than a societal problem, which can shift preferred solutions away from top-down regulation toward personal responsibility or educational interventions. As a result, the motivational link between presumed influence on others and support for governmental or platform-level intervention may be substantially weakened. Importantly, these attributional processes help explain why presumed influence on others alone was insufficient to predict regulatory support.

In this regard, the present study extends the IPMI framework by empirically testing a moderated mediation model that accounts for the interplay between presumed media effects on both the self and others. While previous research has tried to incorporate perceived media effects on self into the IPMI model (Baek et al., 2019), few studies have investigated how such perceptions moderate the relationship between exposure to health misinformation and its presumed influence on others. Addressing this gap, the findings of this study indicate that people’s perceived influence of health misinformation on others functions as a mediator only when they also perceive themselves as susceptible to health misinformation.

This conditional relationship may stem from the nature of health-related issues, which are typically high in personal relevance and perceived risk. Chung and Moon (2016) found that when individuals perceive an issue as closely tied to their wellbeing, their support for media regulation is more strongly influenced by perceived effects on themselves. In the case of health misinformation—whether it promotes vaccine avoidance, ineffective treatments, or conspiracy beliefs—individuals may not only be concerned about their own decisions but also about the misinformed actions of others that could indirectly harm them (Enders et al., 2020). Consequently, health misinformation is often viewed as both a personal and societal threat, which may heighten support for regulatory intervention. Moreover, as Neuwirth and Frederick (2002) proposed, perceiving media influence on both self and others may foster a sense of social consensus about the dangers of misinformation about infectious disease. The present findings align with this perspective, indicating that shared perceptions of vulnerability of health misinformation can serve as a collective motivator for supporting countermeasures against it. In other words, this study suggests when health misinformation is framed as a shared threat to both self and others, public support for regulations is more likely to increase.

The findings of this study provide meaningful guidance for practical application. Specifically, this study suggests that health communicators or policymakers could consider adopting a dual-risk framing approach that highlights both the personal and societal consequences of health misinformation. For instance, they can emphasize potential harm to the self (e.g., compromised treatment decisions) alongside broader public risks (e.g., strain on healthcare systems), thereby enhancing public support for government’s regulatory interventions.

The findings also have implications for social media platforms. The findings suggest that the platforms need to consider implementing interventions that address both individual- and community-level risk perceptions. For instance, misinformation warning labels or credibility indicators could be tailored to underscore not only the societal consequences of misleading content but also its potential to misinform individual users. This dual emphasis may strengthen users’ support for platform-level regulatory measures. In a similar vein, educators and public institutions need to reinforce media literacy initiatives by highlighting personal vulnerability to health misinformation. Instruction should promote critical reflection on how health misinformation may influence individuals’ own health behaviors. Such programs may be especially impactful among populations with high exposure to health misinformation but low perceived susceptibility or risk.

There are several limitations to this study that warrant consideration. First, the analysis is based on cross-sectional survey data. Although numerous studies have supported the causal assumptions of the IPMI model, causal interpretations of the present findings should be made with caution. Future research would benefit from employing longitudinal or experimental designs to more robustly establish causal relationships. Second, although quota sampling was employed at the initial stage based on age, gender, and region, the final sample should not be interpreted as fully representative of the national population of South Korea. Rather, the findings reflect patterns among adult social media users, which constrains their generalizability. In addition, the modest response rate (18.74%) (Wu et al., 2022) raises potential non-response bias concerns, warranting cautious interpretation. Third, the current study relies on self-reported data, asking participants to recall their exposure to health misinformation on social media. However, such self-reports may be subject to recall bias or misclassification, particularly when individuals continue to regard false information as true (Flynn et al., 2017). These limitations are inherent in self-reported measures and may have influenced the interpretation of the data used in this study. Relatedly, exposure to health misinformation was assessed using a single survey item, which may not fully capture variation across platforms or content formats. Nevertheless, the alignment of the findings with established theoretical predictions suggests that this measure demonstrates adequate predictive and construct validity within the present research context (Allen et al., 2022). However, future research should employ multi-item, platform-specific exposure measures to enhance measurement reliability.

Finally, this study was conducted within the specific sociopolitical and cultural context of South Korea during the COVID-19 pandemic. Since perceptions of media influence and regulatory attitudes may vary across cultural and issue contexts, the generalizability of the findings is limited. Future studies should replicate the proposed moderated mediation model across different countries and health misinformation topics.

Despite these limitations, this study contributes to a deeper understanding of the psychosocial mechanisms linking exposure to health misinformation and public support for regulatory actions. By extending the IPMI model, it underscores the critical role of perceived media effects on the self in shaping attitudes toward interventions aimed at curbing health-related misinformation. These findings offer meaningful insights for policymakers, public health communicators, and platform designers seeking to develop more effective and socially sustainable strategies for managing misinformation during health crises. Ultimately, fostering both individual and collective awareness of health misinformation risks may enhance public resilience and support for long-term, sustainable regulatory frameworks.

## Data Availability

The raw data supporting the conclusions of this article will be made available by the authors, without undue reservation.

## Footnotes

1 Kakao-Talk is one of the most popular social media platforms in South Korea.
